# Does bladder outflow obstruction obfuscate the traditional clinical factors that are used to assess the risk of prostate cancer at rapid‐access diagnostic clinics?

**DOI:** 10.1002/bco2.478

**Published:** 2024-12-20

**Authors:** Thomas Philip Cahill, Samuel Joseph Withey, Steve Hazell, Declan Cahill, Netty Kinsella

**Affiliations:** ^1^ School of Medicine University of Liverpool Liverpool UK; ^2^ Department of Urology The Royal Marsden Hospital London UK; ^3^ Department of Radiology The Royal Marsden Hospital London UK; ^4^ Department of Histopathology The Royal Marsden Hospital London UK; ^5^ Translational Oncology and Urology Research King's College London London UK

**Keywords:** bladder outflow obstruction, diagnostics, health services research, prostate cancer, uroflowmetry

## Abstract

**Objectives:**

To understand whether bladder outflow obstruction influences the association between traditional clinical predictive factors, particularly prostate‐specific antigen (PSA) density and clinically significant prostate cancer (csPCa). This will help facilitate effective and evidence‐based triaging of patients in rapid‐access clinics.

**Materials and Methods:**

We retrospectively analysed prospectively collected data from 307 suspected prostate cancer patients who underwent diagnostic biopsy from 2019 to 2023 at a single, high‐volume, specialist cancer centre. Uroflowmetry testing generated two cohorts: patients with bladder outflow obstruction and non‐obstructed patients. The cohort characteristics between the groups were compared and logistic regression analyses were performed to assess associations between clinical predictive factors (age, PSA density, ethnicity, family history, digital rectal examination, urinary symptom severity and magnetic resonance imaging using the PI‐RADS scoring system) and clinically significant prostate cancer (csPCa) on biopsy (defined as International Society of Urological Pathology grade of greater than or equal to two).

**Results:**

The obstructed group (*n* = 80) had significantly larger prostates and worse symptom severity (*p* < 0.05). There was no significant difference between the other predictive factors or csPCa compared to the non‐obstructed (*n* = 227) cohort. Multivariable logistic regression analysis showed age, PSA density, an abnormal digital rectal examination and scoring PI‐RADS 4–5 on magnetic resonance imaging were all significantly associated with csPCa in the non‐obstructed cohort (*p* < 0.05). Contrastingly, only symptom severity and scoring PI‐RADS 5 were significantly associated with csPCa for the obstructed patients (*p* < 0.05).

**Conclusion:**

In the presence of bladder outflow obstruction, traditional predictive variables such as age, PSA density, digital rectal examination and scoring PI‐RADS 4 are not associated with csPCa. This study suggests that using these predictive variables to triage patients in rapid‐access clinics with a patient who has bladder outflow obstruction could lead to the overuse of invasive biopsy.

## 1 INTRODUCTION

The early detection, diagnosis and treatment of prostate cancer is critical to optimise outcomes. To facilitate this, patients can be referred to a urology‐led diagnostic service via a suspected cancer pathway. In the United Kingdom, these services vary between straight‐to‐test triage models and face‐to‐face rapid‐access clinics. In these rapid‐access clinics, a prostate cancer specialist will holistically triage each patient for diagnostic biopsy based on their risk factors and clinical assessment. Risk factors associated with prostate cancer are increasing age, black ethnicity, and a positive family history.^1^

Additionally, clinical assessment typically involves a digital rectal examination (DRE), prostate‐specific antigen (PSA) level testing, investigation of urinary symptom severity and magnetic resonance imaging (MRI**).** ^1^ This imaging provides two critical measurements: a Prostate Imaging‐Reporting and Data System (PI‐RADS) score (a well‐validated method of assessing a patient's risk of prostate cancer)^2^ and the prostate volume (which is used to subsequently calculate PSA density [PSAD]). Finally, urinary symptom severity can be assessed using both self‐reported questionnaires and uroflowmetry. These risk factors and holistic clinical assessment help specialists in rapid‐access clinics to inform risk and triage patients for biopsy.

The National Institute for Health and Care Excellence (NICE) recommend a suspected cancer referral for patients with an abnormally elevated PSA level.^3^ However, patients are also recommended to undergo PSA testing if they are experiencing lower urinary tract symptoms (LUTS) such as voiding difficulty.^3^ These symptoms are often due to bladder outflow obstruction (BOO), related to benign prostatic hyperplasia (BPH)^4^; however, BOO can also arise from prostate cancer, hence the recommended PSA testing.

As PSA levels are significantly associated with BOO^5^ and even negatively associated with prostate cancer when patients have LUTS^6^ there is concern regarding the utility of PSA testing as a predictive factor for prostate cancer in patients with BOO. However, this disrupted association with prostate cancer in the context of BOO is generally unknown for other predictive factors such as PSAD. Understanding this is critical to help facilitate effective and evidence‐based triaging of patients. We hypothesise that the association between PSAD and clinically significant prostate cancer (csPCa) is obfuscated by the presence of BOO.

BOO can be diagnosed using uroflowmetry assessment during triage in the rapid‐access clinics before MRI. However, in straight‐to‐test triage models such as the Rapid Assessment for Prostate Imaging and Diagnosis (RAPID) pathway, patients will be triaged for MRI based on their PSA testing alone and subsequently triaged for biopsy based on their PI‐RADS score and PSAD.^7^ This protocol is believed to provide earlier diagnosis whilst needing less clinical acumen when triaging patients, which is why more clinicians are adopting this practice. However, as straight‐to‐test models do not account for other clinical parameters such as BOO, assessing the relationship between BOO, PSAD and prostate cancer is necessary before this protocol is implemented more widely.

## 2 MATERIALS AND METHODS

### 2.1 Patient population

This retrospective study used prospectively collected data from a cohort of 756 consecutive, biopsy‐naive male patients who were referred to the Royal Marsden Hospital for suspected prostate cancer from July 2019 to April 2023. These patients were triaged by prostate cancer specialists, whereby, those deemed low risk for prostate cancer were discharged (*n* = 346) and those deemed high risk proceeded to biopsy (*n* = 410). The maximum flow rate (Qmax) was used to divide these biopsied patients into two groups: obstructed (patients with BOO) and non‐obstructed. To ensure the validity of the Qmax, all patients with a volume voided of less than 150 ml (*n* = 86) were excluded. Furthermore, those with missing data were also excluded (*n* = 17). Therefore, the final cohort included for analysis was 307 patients.

### 2.2 Uroflowmetry

All patients underwent simple uroflowmetry testing to measure: Qmax, post‐void residual volume and voided volume. The Qmax was used to group patients into either the obstructed cohort (a Qmax of less than or equal to 12.0 ml/s) or the non‐obstructed cohort (a Qmax of greater than 12.0 ml/s). To calculate the Qmax all patients voided into a flowmeter. Additionally, patients underwent a bladder scan to assess post‐void residual volume. To ensure a valid result, a minimum voided volume of 150 ml was required.

Despite pressure‐flow urodynamic studies being the gold standard for diagnosing BOO, it has been suggested that Qmax measured using uroflowmetry is an acceptable adjunct in certain situations.^8^ Therefore, uroflowmetry was chosen to stratify patients into the two groups in this study as it provides objective, repeatable results and the process is tolerable for patients and deliverable in the outpatient setting.

### 2.3 Magnetic resonance imaging

Multiparametric MRI (mp‐MRI) which comprised of T2‐weighted imaging, diffusion‐weighted imaging (DWI) and dynamic post‐contrast fat‐saturated T1‐weighted imaging (DCE) was performed. Patients with contraindications to intravenous contrast administration underwent biparametric MRI. The prostate volume was calculated (as recommended in PI‐RADS v2.1) using the formula for calculating the volume of an ellipsoid: the product of tridirectional measurements and 0.52.^9^ Each MRI was interpreted by one of five expert readers: each having reported at least 1000 cases.^10^ A PI‐RADS score was assigned to each lesion, and then to the overall gland corresponding to the highest‐scoring lesion. This whole gland score was recorded and used for analysis. The PI‐RADS scoring system is scored from one to five and is used to denote different likelihoods of csPCa being present. A score of one indicates a very low likelihood, two indicates a low likelihood, three indicates an intermediate likelihood, four represents a high likelihood and five represents a very high likelihood of csPCa being present.^9^

### 2.4 Variable assessment

The outcome variable for this retrospective analysis was csPCa on biopsy. All 307 patients had transperineal prostate biopsies under local anaesthetic, using the Koelis system.^11^ MRI fusion technology^12^ was used to target lesions which involved taking 2–3 cores per lesion with additional systematic cores from the peripheral zone. If there was no lesion, biopsies were performed systematically with 10 cores for a prostate gland volume of less than 25 ml and 12 cores for a prostate gland of greater than or equal to 25 ml.

After collecting these tissue cores via biopsy, they were processed with a maximum of two cores per cassette and a minimum of three levels examined per cassette. Grading was performed by the same specialist uropathologist in accordance with the International Society of Urological Pathology (ISUP) guidelines (2014)^13^ and the Royal College of Pathologists Prostate Reporting guidelines (2016).^14^ Individual assessments of the overall and highest ISUP grade (and Gleason grade) were performed at each biopsy site and over the whole biopsy set. Subsequently, an ISUP grade was recorded and csPCa was defined as an ISUP grade of greater or equal to two.

The exposure variables measured in this retrospective analysis were age (at the time of biopsy), ethnicity, family history (of prostate cancer), DRE findings, PSA levels, prostate volume, PSAD, PI‐RADS score and urinary symptom severity. Ethnicity (white, black or other), family history of prostate cancer (yes or no) and symptom severity were all self‐reported by the patients at the rapid‐access clinic. Symptom severity was measured using the International Prostate Symptom Score (IPSS): higher scores indicate more severe LUTS. DRE was assessed by the diagnostics team at the rapid‐access clinic and reported as either abnormal or benign. Prostate volume and PI‐RADS score were obtained from MRI, as previously described. PSA was recorded at referral and validated by a repeat test which was then used to calculate PSAD (recorded PSA value divided by the prostate volume).

### 2.5 Analysis

Statistical analysis was performed using R Studio (version 4.3.1, 2023), with significance set at *p* < 0.05. Cohort characteristics between the two groups were compared. Categorical variables (ethnicity, family history, DRE, PI‐RADS and csPCa) were compared using the Chi‐Squared test. Continuous variables underwent preliminary assessment for parametric testing suitability using the Shapiro–Wilk test. Subsequently, continuous variables that were suitable (age) were compared using the Welch Two Sample t‐test and those unsuitable (PSA, prostate volume, PSAD and IPSS) were compared using the Wilcoxon Rank Sum Test.

The association between the exposure variables with csPCa were analysed using univariate and multivariable logistic regression analysis: significance was considered if *p* < 0.05. Prior to performing analysis, all categorical variables were “dummy” encoded and the PSAD data was multiplied by a factor of 10 to facilitate easier clinical interpretation. Notably, it is generally accepted that age, ethnicity, family history, DRE and PI‐RADS should be used in the assessment of prostate cancer; therefore, all were included in the multivariable analysis regardless of their significance in the univariate testing.^1^ Furthermore, as IPSS has been shown to be significantly lower in those with prostate cancer compared to those without prostate cancer, this was also to be included in the multivariable analysis.^15^

### 2.6 Ethical statement

This retrospective cohort study was conducted following institutional review board waiver of consent (RMH/SE1100).

## 3 RESULTS

### 3.1 Cohort characteristics

In total, 307 male patients who underwent MRI and proceeded to biopsy were included in this study. These patients were grouped based on the presence or absence of BOO. The characteristics of these two groups were analysed and differences were compared (Table 1): obstructed (*n* = 80) and non‐obstructed (*n* = 227). There was no significant difference in age, PSA, PSAD, ethnicity, family history, DRE findings or PI‐RADS score between the obstructed and non‐obstructed cohorts. However, the obstructed cohort had significantly larger prostates and worse symptom severity (measured using the IPSS) compared to the non‐obstructed group. These findings are expected for patients with BOO. Importantly, there was no significant difference between the two cohorts in the outcome variable: csPCa on biopsy (Table 1).

**TABLE 1:** Characteristics of the two cohorts of patients

Variable		Obstructed (*n* = 80)	Non‐obstructed (*n* = 227)	*p*‐value
Mean age (IQR) /years		66.15 (61.00–71.25)	64.46 (58.00–71.00)	0.092
Median PSA (IQR) /ng/ml		6.78 (5.15–10.45)	6.20 (4.30–9.97)	0.068
Median prostate volume (IQR) /ml		44.50 (36.00–58.25)	36.00 (29.00–50.50)	**<0.001**
Median PSAD (IQR) /ng/ml^2^		0.148 (0.111–0.255)	0.155 (0.115–0.265)	0.640
Ethnicity	White	78.75% (*n* = 63)	73.57% (*n* = 167)	0.494
	Black	6.25% (*n* = 5)	10.57% (*n* = 24)
	Other	15.00% (*n* = 12)	15.86% (*n* = 36)
Family history of prostate cancer	No	71.25% (*n* = 57)	70.48% (*n* = 160)	1.00
	Yes	28.75% (*n* = 23)	29.52% (*n* = 67)
Abnormal DRE	No	40.00% (*n* = 32)	36.12% (*n* = 82)	0.629
	Yes	60.00% (*n* = 48)	63.88% (*n* = 145)
Median IPSS (IQR)		13.00 (7.00–20.25)	10.00 (5.00–15.00)	**<0.01**
PI‐RADS score	2	8.75% (*n* = 7)	10.57% (*n* = 24)	0.651
	3	21.25% (*n* = 17)	25.55% (*n* = 58)
	4	46.25% (*n* = 37)	38.33% (*n* = 87)
	5	23.75% (*n* = 19)	25.55% (*n* = 58)
Clinically significant prostate cancer on biopsy		61.3% (*n* = 49)	63.0% (*n* = 143)	0.886

Abbreviations: IQR = Interquartile Range; PSA = Prostate‐Specific Antigen; PSAD = PSA Density; DRE = Digital Rectal Examination; IPSS = International Prostate Symptom Score; PI‐RADS = Prostate Imaging‐Reporting and Data System. Significance: *p* < 0.05.

### 3.2 Univariate analysis

Next, univariate logistic regression was used to analyse any association between nine exposure variables with csPCa both in the presence and absence of obstruction (Table 2). In the absence of obstruction, the univariate analysis suggested that older age (OR 1.08; 95% CI 1.04–1.11), higher PSA levels (OR 1.10; 95% CI 1.03–1.16), a smaller prostate volume (OR 0.98; 95% CI 0.96–0.99), a higher PSAD (OR 1.93; 95% CI 1.42–2.62), an abnormal DRE (OR 3.31; 95% CI 1.87–5.84) and PI‐RADS 4 (OR 8.44; 95% CI 2.85–24.99) or PI‐RADS 5 (OR 51.30; 95% CI 12.46–211.16) were all significantly associated with higher odds of csPCa. Conversely, in the presence of obstruction, only older age (OR 1.09; 95% CI 1.02–1.18), a higher PSAD (OR 1.62; 95% CI 1.04–2.52), a lower IPSS (OR 0.93; 95% CI 0.88–0.99) and PI‐RADS 5 (OR 45.00; 95% CI 3.35–603.96) were significantly associated with csPCa on univariate analysis.

**TABLE 2:** Results following univariate logistic regression for both the obstruct and non‐obstructed cohorts

Exposure Variable		Obstructed (*n* = 80)		Non‐obstructed (*n* = 227)	
		Odds Ratio (95% CI)	*p*‐value	Odds Ratio (95% CI)	*p*‐value
Age (years)		1.09 (1.02–1.18)	**0.011**	1.08 (1.04–1.11)	**<0.001**
PSA (ng/ml)		1.07 (0.99–1.17)	0.102	1.10 (1.03–1.16)	**<0.01**
Prostate volume (ml)		0.98 (0.96–1.00)	0.122	0.98 (0.96–0.99)	**<0.001**
PSADx10 (ng/ml^2^)		1.62 (1.04–2.52)	**0.034**	1.93 (1.42–2.62)	**<0.001**
Ethnicity	White	1.00 (Reference)		1.00 (Reference)	
	Black	0.80 (0.12–5.18)	0.819	0.65 (0.27–1.53)	0.3206
	Other	0.38 (0.10–1.35)	0.136	0.86 (0.41–1.80)	0.687
Family history of prostate cancer	No	1.00 (Reference)		1.00 (Reference)	
	Yes	1.27 (0.46–3.48)	0.644	0.75 (0.42–1.35)	0.334
Abnormal DRE	No	1.00 (Reference)		1.00 (Reference)	
	Yes	1.42 (0.57–3.54)	0.454	3.31 (1.87–5.84)	**<0.001**
IPSS		0.93 (0.88–0.99)	**0.014**	0.99 (0.95–1.03)	0.552
PIRADS	2	1.00 (Reference)		1.00 (Reference)	
	3	1.36 (0.20–9.28)	0.751	2.68 (0.88–8.18)	0.083
	4	4.11 (0.70–24.10)	0.118	8.44 (2.85–24.99)	**<0.001**
	5	45.00 (3.35–603.96)	**<0.01**	51.30 (12.46–211.16)	**<0.001**

Abbreviations: PSA = Prostate‐Specific Antigen; PSAD = PSA Density; DRE = Digital Rectal Examination; IPSS = International Prostate Symptom Score; PI‐RADS = Prostate Imaging‐Reporting and Data System. Significance: *p* < 0.05.

Age, PSAD, ethnicity, family history, DRE findings, IPSS and PI‐RADS score would be used in the multivariable analysis due to their known association with prostate cancer. However, PSA and prostate volume were not used in the multivariable analysis. The reasoning behind this is multifaceted. Firstly, PSAD is derived from PSA divided by prostate volume and therefore likely to be highly correlated. Secondly, in the univariate analysis, PSAD was more significantly associated with csPCa compared to both PSA and prostate volume for both the obstructed and non‐obstructed cohorts. Thirdly, it is now widely accepted that PSAD is of more clinical use compared to PSA or prostate volume alone.

### 3.3 Multivariable analysis

Multivariable analysis next assessed the association between age, PSAD, ethnicity, family history, DRE findings, IPSS and PI‐RADS score with csPCa in the presence and absence of obstruction. In the absence of obstruction, the multivariable analysis showed that older age (OR 1.05; 95% CI 1.01–1.10), a higher PSAD (OR 1.90; 95% CI 1.35–2.65), an abnormal DRE (OR 2.16; 95% CI 1.07–4.33), PI‐RADS 4 (OR 10.17; 95% CI 2.92–35.36) and PI‐RADS 5 (OR 24.16; 95% CI 5.11–114.32) were all significantly associated with higher odds of csPCa. However, in the presence of obstruction, only PI‐RADS 5 (OR 92.37; 95% CI 3.85–2213.49) and IPSS (OR 0.89; 95% CI 0.81–0.97) were significantly associated with csPCa on multivariable analysis. The significance of the association of age, PSAD, DRE and PI‐RADS 4 with csPCa was seemingly dependent on the obstruction status of the patient. Additionally, there was no association observed between ethnicity, family history or PI‐RADS 3 with csPCa for both cohorts. Finally, IPSS was not found to be associated with csPCa in the absence of obstruction, however, in the presence of obstruction there was a significant association, indicating the greater the symptom severity, the lower the odds of csPCa.

## 4 DISCUSSION

A total of 307 patients who underwent both MRI and subsequent biopsy were included in this study. Basic uroflowmetry testing stratified patients based on the presence or absence of BOO, resulting in two groups: obstructed (*n* = 80) and non‐obstructed (*n* = 227). The obstructed cohort was smaller, and had significantly worse symptom severity and larger prostates; however, there were no other significant differences between the cohort characteristics. The multivariable logistic regression analysis demonstrated that the association between some clinical parameters and csPCa was related to the presence or absence of BOO. The primary finding of this study was that increasing PSAD (OR 1.90; 95% CI 1.35–2.65) was significantly associated with csPCa in the non‐obstructed cohort when adjusted for age, ethnicity, family history, DRE, PI‐RADS score and IPSS: this was not observed in the obstructed cohort (Table 3). This finding indicates that using PSAD alone to stratify a patient's risk for prostate cancer when they have BOO could be inappropriate and lead to the overuse of invasive diagnostic biopsies.

**TABLE 3:** Results of the multivariable analysis performed on both cohorts of patients

Exposure Variable		Obstructed (*n* = 80)		Non‐obstructed (*n* = 227)	
		Odds Ratio (95% CI)	*p*‐value	Odds Ratio (95% CI)	*p*‐value
Age (years)		1.08 (0.97–1.19)	0.143	1.05 (1.01–1.10)	**0.024**
PSADx10 (ng/ml^2^)		1.80 (0.89–3.64)	0.101	1.90 (1.35–2.65)	**<0.001**
Ethnicity	White	1.00 (Reference)		1.00 (Reference)	
	Black	3.89 (0.29–52.71)	0.306	1.27 (0.39–4.11)	0.689
	Other	0.96 (0.16–5.75)	0.964	1.24 (0.48–3.24)	0.654
Family history of prostate cancer	No	1.00 (Reference)		1.00 (Reference)	
	Yes	3.04 (0.69–3.43)	0.142	0.87 (0.41–1.87)	0.722
Abnormal DRE	No	1.00 (Reference)		1.00 (Reference)	
	Yes	1.45 (0.37–5.74)	0.595	2.16 (1.07–4.33)	**0.031**
PI‐RADS score	2	1.00 (Reference)		1.00 (Reference)	
	3	3.50 (0.34–36.55)	0.295	3.28 (0.92–11.70)	0.068
	4	7.60 (0.88–65.48)	0.065	10.17 (2.92–35.36)	**<0.001**
	5	92.37 (3.85–2213.49)	**<0.01**	24.16 (5.11–114.32)	**<0.001**
IPSS		0.89 (0.81–0.97)	**<0.01**	1.00 (0.96–1.05)	0.939

Abbreviations: PSA = Prostate‐Specific Antigen; PSAD = PSA Density; DRE = Digital Rectal Examination; IPSS = International Prostate Symptom Score; PI‐RADS = Prostate Imaging‐Reporting and Data System. Significance: *p* < 0.05.

Furthermore, in the non‐obstructed cohort both a PI‐RADS score of 4 and 5 were significantly associated with csPCa when adjusted for age, PSAD, ethnicity, family history, DRE and IPSS. However, in the obstructed cohort, only PI‐RADS 5 was significantly associated when adjusted for the same variables (Table 3). This loss of association between PI‐RADS 4 and csPCa in the presence of obstruction may indicate that subcategorising PI‐RADS 4 patients based on their obstruction status could prevent the overuse of diagnostic biopsies. Previous research has outlined the utility of subcategorising patients based on MRI features^16^ and we believe extending this research to assess the use of uroflowmetry for further stratification of patients within the PI‐RADS scoring system would be beneficial.

Additionally, an abnormal DRE (OR 2.16; 95% CI 1.07–4.33) was significantly associated with csPCa in the absence of obstruction when adjusted for age, PSAD, ethnicity, family history, PI‐RADS score and IPSS: this was not observed in the obstructed cohort. This finding may explain the lack of association between DRE and csPCa in previous research where uroflowmetry to identify obstruction was not performed.^17^ By potentially including patients with BOO in the whole cohort analysis looking for an association between DRE and csPCa, this could falsely give the impression that DRE is not associated with csPCa. However, the present study suggests a significant association in the absence of BOO. This finding could have implications in the clinical triage of patients and aid in the interpretation of both previous and future clinical research that assesses DRE utility.

Conversely, in the obstructed cohort, IPSS (OR 0.89; 95% CI 0.81–0.97) was found to be significantly associated with csPCa when adjusted for age, PSAD, ethnicity, family history, DRE and PI‐RADS. This was an interesting and unexpected finding as IPSS was not significantly associated with csPCa in the non‐obstructed cohort. This finding suggests that in the obstructed cohort, the higher the IPSS (and therefore worse self‐reported symptom severity) the lower the odds of csPCa: indicating that benign causes of BOO are more likely to cause worse symptom severity compared to prostate cancer. This is useful clinically as following the exclusion of prostate cancer, this can be used to help reassure patients that despite having bothersome symptoms the evidence suggests the underlying cause is more likely benign.

The observations of the present study provide insights into the utility of uroflowmetry in diagnostic triage. However, they also provide insight into the underlying physiological causality behind the previously described lack of association between PSA and csPCa in patients with BOO (Table 2). There are currently two working explanations for this. Firstly, in this study, the obstructed cohort had significantly larger prostates (Table 1). Larger prostates are associated with elevated PSA even in the absence of prostate cancer.^18^ This makes it challenging to differentiate between benign causes of BOO and prostate cancer as PSA levels are raised in both conditions. However, the insignificant association between PSAD and csPCa (Table 3) makes this theory less plausible as by definition the PSA has been adjusted for the prostate volume. Secondly, individuals with obstruction typically experience high voiding pressure.^19^ This has been suggested to cause intra‐prostatic urinary reflux and local chemical prostatitis, contributing to an elevation in PSA in all obstructed patients regardless of a benign or malignant cause.^20^ We believe this study supports this hypothesis as PSAD was not significantly associated with csPCa in the obstructed cohort when adjusting for other variables (Table 3). This indicates that the underlying reason why PSA is not predictive of prostate cancer in patients with LUTS is not due to the prostate volume and that patients with BOO may have a falsely raised PSA due to the high pressure voiding and not due to prostate cancer risk.

Despite the results of this study being noteworthy, there are some limitations. Firstly, the study had specific inclusion criteria, such as needing a volume voided of greater or equal to 150 ml on uroflowmetry. This resulted in a small sample size, particularly in the obstructed cohort (*n* = 80). This is important to note as it resulted in the reference groups for the categorical variables being small: only seven patients were PI‐RADS 2 and obstructed which may explain the lack of statistical significance for the association between PI‐RADS 4 and csPCa in this cohort (Table 2). Secondly, there were no patients who scored PI‐RADS 1 in this study. As these patients are deemed to have a very low likelihood of csPCa, they did not receive a biopsy^9^ and as such could not be included in this study. Thirdly, this retrospective study used data that was collected from only one specialist centre that routinely performs uroflowmetry assessment. Therefore, the findings of this study may not be transferable to other rapid‐access clinics that do not routinely perform uroflowmetry assessment. Fourthly, it is important to note that most patients included in this study were reported as ethnically white: 78.75% and 73.57% in the obstructed and non‐obstructed cohorts, respectively. It would have been interesting to have a larger cohort of those reported as ethnically black as there appeared to be a weaker relationship between black ethnicity and csPCa in the non‐obstructed cohort compared to the obstructed cohort; however, this was not significant. Finally, despite previous evidence suggesting a relatively high sensitivity for uroflowmetry detecting BOO,^21^ caution is needed in interpreting these results and confirmatory future research that diagnoses BOO using the gold standard, pressure‐flow urodynamic studies is key.^8^

## 5 CONCLUSION

This study has provided preliminary evidence of the utility of performing uroflowmetry testing to stratify patients into obstructed and non‐obstructed cohorts in rapid‐access clinics. This study suggests there is a weaker association between PSAD, age, an abnormal DRE and the PI‐RADS scoring system and csPCa in the presence of obstruction. The results highlight a limitation to current straight‐to‐test pathways and indicate a potential use for uroflowmetry testing, not necessarily as a diagnostic tool but to improve risk stratification by improving the reliability of predictive factors. This could be used to help prevent the overuse of invasive diagnostics in this group of patients.

## AUTHOR CONTRIBUTIONS

**Thomas Philip Cahill:** Data collation; data analysis; manuscript preparation. **Samuel Joseph Withey:** Data analysis; manuscript preparation; supervision. **Steve Hazell:** Pathology analysis; manuscript preparation. **Declan Cahill:** Data analysis; supervision; manuscript preparation. **Netty Kinsella:** Supervision; data analysis; manuscript preparation.

## CONFLICT OF INTEREST STATEMENT

The authors of this manuscript have no conflicts of interest to declare and have completed the ICMJE conflicts of interest disclosure form.

## References

[1] National Institute for Health and Care Excellence . Prostate cancer: diagnosis and management [internet]. NICE; 2019 [updated 2021 Dec; cited 2024 Feb 14]. (NICE guideline [NG131]). https://www.nice.org.uk/guidance/ng131/chapter/Recommendations 35263066

[2] Weinreb JC , Barentsz JO , Choyke PL , Cornud F , Haider MA , Macura KJ , et al. PI‐RADS prostate imaging ‐ reporting and data system: 2015, version 2. Eur Urol. 2016;69(1):16–40. 10.1016/j.eururo.2015.08.052 26427566 PMC6467207

[3] National Institute for Health and Care Excellence . Suspected cancer: recognition and referral [internet]. NICE; 2015 [updated 2023 Oct; cited 2024 Feb 14]. (NICE guideline [NG12]). Available from: https://www.nice.org.uk/guidance/ng12/chapter/Recommendations-organised-by-site-of-cancer#urological-cancers

[4] Abdelmoteleb H , Jefferies ER , Drake MJ . Assessment and management of male lower urinary tract symptoms (LUTS). Int J Surg. 2016;25:164–171. 10.1016/j.ijsu.2015.11.043 26654899

[5] Laniado ME , Ockrim JL , Marronaro A , Tubaro A , Carter SS . Serum prostate‐specific antigen to predict the presence of bladder outlet obstruction in men with urinary symptoms. BJU Int. 2004;94(9):1283–1286. 10.1111/j.1464-410X.2004.05158.x 15610106

[6] Collin SM , Metcalfe C , Donovan J , Lane JA , Davis M , Neal D , et al. Associations of lower urinary tract symptoms with prostate‐specific antigen levels, and screen‐detected localized and advanced prostate cancer: a case‐control study nested within the UK population‐based ProtecT (prostate testing for cancer and treatment) study. BJU Int. 2008;102(10):1400–1406. 10.1111/j.1464-410X.2008.07817.x 18540932

[7] Eldred‐Evans D , Connor MJ , Bertoncelli Tanaka M , Bass E , Reddy D , Walters U , et al. The rapid assessment for prostate imaging and diagnosis (RAPID) prostate cancer diagnostic pathway. BJU Int. 2023;131(4):461–470. 10.1111/bju.15899 36134435

[8] Reynard JM , Yang Q , Donovan JL , Peters TJ , Schafer W , de la Rosette JJ , et al. The ICS‐‘BPH’ study: uroflowmetry, lower urinary tract symptoms and bladder outlet obstruction. Br J Urol. 1998;82(5):619–623. 10.1046/j.1464-410x.1998.00813.x 9839573

[9] Scott R , Misser SK , Cioni D , Neri E . PI‐RADS v2.1: what has changed and how to report. *SA* . J Radiol. 2021;25(1):2062. 10.4102/sajr.v25i1.2062 PMC8252188 34230862

[10] de Rooij M , Israel B , Tummers M , Ahmed HU , Barrett T , Giganti F , et al. ESUR/ESUI consensus statements on multi‐parametric MRI for the detection of clinically significant prostate cancer: quality requirements for image acquisition, interpretation and radiologists' training. Eur Radiol. 2020;30(10):5404–5416. 10.1007/s00330-020-06929-z 32424596 PMC7476997

[11] Covin B , Roumiguie M , Quintyn‐Ranty ML , Graff P , Khalifa J , Aziza R , et al. Refining the risk‐stratification of transrectal biopsy‐detected prostate cancer by elastic fusion registration transperineal biopsies. World J Urol. 2019;37(2):269–275. 10.1007/s00345-018-2459-4 30145777

[12] Ukimura O , Desai MM , Palmer S , Valencerina S , Gross M , Abreu AL , et al. 3‐Dimensional elastic registration system of prostate biopsy location by real‐time 3‐dimensional transrectal ultrasound guidance with magnetic resonance/transrectal ultrasound image fusion. J Urol. 2012;187(3):1080–1086. 10.1016/j.juro.2011.10.124 22266005

[13] Epstein JI , Egevad L , Amin MB , Delahunt B , Srigley JR , Humphrey PA , et al. The 2014 International Society of Urological Pathology (ISUP) consensus conference on Gleason grading of prostatic carcinoma: definition of grading patterns and proposal for a new grading system. Am J Surg Pathol. 2016;40(2):244–252. 10.1097/PAS.0000000000000530 26492179

[14] Oxley J , Varma M , Berney D . Standards and datasets for reporting cancers dataset for histopathology reports for prostatic carcinoma [internet]. Royal College of Pathologists; 2016 [cited 2024 June 14]. Available from: https://www.rcpath.org/static/8cc88604-2c8d-4df4-a99542df41c102af/G048-ProstateDataset-Jun16.pdf

[15] Bruno SM , Falagario UG , d'Altilia N , Recchia M , Mancini V , Selvaggio O , et al. PSA density help to identify patients with elevated PSA due to prostate cancer rather than intraprostatic inflammation: a prospective single center study. Front Oncol. 2021;11:693684. 10.3389/fonc.2021.693684 34094990 PMC8173030

[16] Messina E , Pecoraro M , Laschena L , Bicchetti M , Proietti F , Ciardi A , et al. Low cancer yield in PI‐RADS 3 upgraded to 4 by dynamic contrast‐enhanced MRI: is it time to reconsider scoring categorization? Eur Radiol. 2023;33(8):5828–5839. 10.1007/s00330-023-09605-0 37045981 PMC10326099

[17] Jahnen M , Hausler T , Meissner VH , Ankerst DP , Kattan MW , Sauter A , et al. Predicting clinically significant prostate cancer following suspicious mpMRI: analyses from a high‐volume center. World J Urol. 2024;42(1):290. 10.1007/s00345-024-04991-6 38702557 PMC11068682

[18] Roehrborn CG , Boyle P , Gould AL , Waldstreicher J . Serum prostate‐specific antigen as a predictor of prostate volume in men with benign prostatic hyperplasia. Urology. 1999;53(3):581–589. 10.1016/S0090-4295(98)00655-4 10096388

[19] Griffiths D , Hofner K , van Mastrigt R , Rollema HJ , Spangberg A , Gleason D . Standardization of terminology of lower urinary tract function: pressure‐flow studies of voiding, urethral resistance, and urethral obstruction. International continence society subcommittee on standardization of terminology of pressure‐flow studies. NeurourolUrodyn. 1997;16(1):1–18. 10.1002/(SICI)1520-6777(1997)16:1<1::AID-NAU1>3.0.CO;2-I 9021786

[20] Cormio L , Lucarelli G , Selvaggio O , di Fino G , Mancini V , Massenio P , et al. Absence of bladder outlet obstruction is an independent risk factor for prostate cancer in men undergoing prostate biopsy. Medicine (Baltimore). 2016;95(7):e2551. 10.1097/MD.0000000000002551 26886598 PMC4998598

[21] Oelke M , Höfner K , Jonas U , de la Rosette JJ , Ubbink DT , Wijkstra H . Diagnostic accuracy of noninvasive tests to evaluate bladder outlet obstruction in men: detrusor wall thickness, uroflowmetry, postvoid residual urine, and prostate volume. Eur Urol. 2007;52(3):827–835. 10.1016/j.eururo.2006.12.023 17207910

